# First steps in using machine learning on fMRI data to predict intrusive memories of traumatic film footage

**DOI:** 10.1016/j.brat.2014.07.010

**Published:** 2014-11

**Authors:** Ian A. Clark, Katherine E. Niehaus, Eugene P. Duff, Martina C. Di Simplicio, Gari D. Clifford, Stephen M. Smith, Clare E. Mackay, Mark W. Woolrich, Emily A. Holmes

**Affiliations:** a University Department of Psychiatry, Warneford Hospital, University of Oxford, United Kingdom; b Institute of Biomedical Engineering, Department of Engineering Science, University of Oxford, United Kingdom; c FMRIB Centre, Nuffield Department of Clinical Neurosciences, John Radcliffe Hospital, University of Oxford, United Kingdom; d Medical Research Council Cognition and Brain Sciences Unit, 15 Chaucer Road, Cambridge CB2 7EF, United Kingdom; e Oxford Centre for Human Brain Activity (OHBA), Department of Psychiatry, Warneford Hospital, University of Oxford, United Kingdom; f Department of Clinical Neuroscience, Karolinska Institutet, Stockholm, Sweden

**Keywords:** Intrusive memories, Trauma, Flashback, MVPA, Machine learning, Functional magnetic resonance imaging, Mental imagery

## Abstract

After psychological trauma, why do some only some parts of the traumatic event return as intrusive memories while others do not? Intrusive memories are key to cognitive behavioural treatment for post-traumatic stress disorder, and an aetiological understanding is warranted. We present here analyses using multivariate pattern analysis (MVPA) and a machine learning classifier to investigate whether peri-traumatic brain activation was able to *predict* later intrusive memories (i.e. before they had happened). To provide a methodological basis for understanding the context of the current results, we first show how functional magnetic resonance imaging (fMRI) *during* an experimental analogue of trauma (a trauma film) via a prospective event-related design was able to capture an individual's later intrusive memories. Results showed widespread increases in brain activation at encoding when viewing a scene in the scanner that would later return as an intrusive memory in the real world. These fMRI results were replicated in a second study. While traditional mass univariate regression analysis highlighted an association between brain processing and symptomatology, this is not the same as prediction. Using MVPA and a machine learning classifier, it was possible to predict later intrusive memories across participants with 68% accuracy, and within a participant with 97% accuracy; i.e. the classifier could identify out of multiple scenes those that would later return as an intrusive memory. We also report here brain networks key in intrusive memory prediction. MVPA opens the possibility of decoding brain activity to reconstruct idiosyncratic cognitive events with relevance to understanding and predicting mental health symptoms.

## Highlights

• Why only some moments within a trauma intrude while others do not is unclear.
• Neuroimaging may provide further clues as to why this is the case.
• Multivariate pattern analysis, a recent neuroimaging analysis tool, was able to predict intrusive memories.
• Those brain networks involved in intrusive memory prediction are presented.
• Multivariate pattern analysis may inform future innovation in mental health.

## Introduction

The focus of the current paper is on using neuroimaging to understand the development of intrusive memories of trauma, that is “recurrent, involuntary and intrusive distressing memories of the traumatic event” (The Diagnostic and Statistical Manual of Mental Disorders, 5th ed.; DSM-5; American Psychiatric Association, 2013). Intrusive memories are a hallmark symptom from the re-experiencing cluster of Post-Traumatic Stress Disorder (PTSD). They have previously been defined as involuntary mental images that occur in a waking state (Frankel, 1994, Jones et al., 2003). Thus, key features of intrusive memories are that they are involuntary rather than deliberately retrieved, i.e. apparently spontaneous (Kvavilashvili, 2014); include perceptual aspects of the traumatic event, i.e. involve mental imagery rather than only verbal thought (Holmes, Grey, & Young, 2005); are in line with episodic and memory recall more broadly (Conway, 2001), and have distressing, i.e. emotional content (Hackmann, Ehlers, Speckens, & Clark, 2004). For example, after a motor vehicle accident, seeing scaffolding smashing through the car windscreen (see Grey and Holmes, 2008, Holmes et al., 2005 for further examples). In their most extreme form, re-experiencing symptoms can present as so-called dissociative ‘flashbacks’ where patients relive past events as if they are happening in the present (American Psychiatric Association, 2013). In contrast, during the experience of an intrusive memory the past events are spontaneously remembered while awareness of the present is maintained.

Due to the nature of this special issue, “*How neuroscience informs behavioural treatment*” within *Behaviour Research and Therapy*, we appreciate that many readers may not have a detailed understanding of neuroimaging terms and techniques. We therefore present a slightly longer than normal introduction to guide the reader through the steps taken before performing the main predictive analysis presented here. We first describe our initial study using traditional neuroimaging analysis techniques (Bourne, Mackay, & Holmes, 2013) and its subsequent replication (Clark, Holmes, Woolrich, & Mackay, submitted for publication). We then introduce the ideas of multivariate pattern analysis (MVPA) and machine learning, before next describing how we utilised these techniques in the current experiment. The aim of this is to provide a methodological basis for understanding the context of the current results and show that these findings are both replicable and reliable. We believe that by using neuroimaging techniques in addition to behavioural, cognitive and psychophysiological experiments we may be able to identify those neural and cognitive functions that are critical for intrusive memory formation. Understanding how intrusive memories are formed from multiple perspectives may allow future work to improve the ability to refine treatments which target the underlying mechanisms of intrusive memory (i.e. symptom) development. Indeed, by gaining the most comprehensive understanding of differences at an individual level, we may be able to open future possibilities of early screening for risk of PTSD, as well as the development of preventative approaches in the immediate aftermath of trauma and for targeted early interventions.

We also note that many different approaches to machine learning and MVPA are evolving, including (but not limited to) Random Forest Theory (Breiman, 2001), Graph theory (Power et al., 2011, Sporns, 2014) and Representational Similarity Analysis (Kriegeskorte, Mur, & Bandettini, 2008), in addition to that used here, a Support Vector Machine classifier (Pereira, Mitchell, & Botvinick, 2009). The current work represents only first steps in applying neuroimaging techniques to understand the neural impact of witnessing trauma and to inform behavioural treatment. We finish by exploring how such techniques might have implications for future cognitive behavioural therapy.

### Intrusive memories and PTSD

Most people will experience a traumatic event during the course of their lifetime and a significant minority will go on to develop PTSD (Breslau et al., 1998, Kessler et al., 1995). We have successful treatments for the full blown disorder, those recommended by clinical guidelines (e.g. National Institute for Health and Clinical Excellence, 2005) are Cognitive Behavioural Therapy (CBT; e.g. Ehlers and Clark, 2000, Foa and Rothbaum, 1998) and Eye Movement Desensitisation and Reprocessing (EMDR; Shapiro, 1995). However, satisfactory preventative treatments against PTSD development are lacking (Roberts, Kitchiner, Kenardy, & Bisson, 2009). A greater understanding of the brain mechanisms that lead to the development of intrusive memories may help guide effective preventative interventions for the early aftermath of trauma.

We know little, in particular in terms of neuroscience, about why only certain events within a trauma return as intrusive memories when others do not. Processing at the time of trauma (peri-traumatic) is implicated in PTSD development (e.g. Brewin, 2014, Ehlers and Clark, 2000, Ozer et al., 2003). Additionally, experimental findings implicate heightened emotional processing at the time of the event in intrusive memory development (Clark et al., 2013, Clark et al., 2014). Interestingly, dissociation, defined within the DSM 5 as “a disruption of and/or discontinuity in the normal integration of consciousness, memory, identity, emotion …” (American Psychiatric Association, 2013, p. 291), can be a reaction to extreme emotion, and peri-traumatic dissociation has also been associated with intrusive memory formation (e.g. Daniels et al., 2012, Holmes et al., 2004). Seminal work on ‘flashbulb’ memories, defined as ‘memories for the circumstances in which one first learned of a very surprising and consequential (or emotionally arousing) event’ (Brown & Kulik, 1977) may also illuminate some of the mechanisms involved in intrusive memory formation. While flashbulb memories are a distinct phenomenon (and not exclusive to trauma, but part of autobiographical memory more generally), they may lie on a continuum with intrusive memories. Research suggests that memories that end up as flashbulb memories are psychophysiologically arousing, personally salient and unexpected and sudden (Brown & Kulik, 1977). Indeed, psychophysiology has been associated with intrusive memory development; at the time of viewing a specific film scene that is later recalled as an intrusive memory, heart rate has been shown to drop in comparison to the rest of film viewing (Chou et al., 2014, Holmes et al., 2004). Understanding the neural processes involved in intrusive memory formation adds another level of comprehension of this complex phenomenon.

### Neuroimaging and established PTSD

The majority of studies using neuroimaging to investigate PTSD have done so once symptoms are already established in patients (Francati et al., 2007, Hughes and Shin, 2011, Pitman et al., 2012). Neurocircuitry models suggest that PTSD is characterised by reduced activity in the ventromedial prefrontal cortex, which is associated with decision making and emotional response inhibition, and increased activation in the amygdala and other limbic areas, which are associated with emotional processing (e.g. Rauch et al., 2006, Rauch et al., 1998). A further recent model suggests that abnormalities in the amygdala and dorsal anterior cingulate cortex are pre-disposing, while abnormal interactions between the hippocampus and ventromedial prefrontal cortex arise after developing PTSD (Admon, Milad, & Hendler, 2013). While informative for understanding PTSD as a whole, these studies cannot tell us specifically about intrusive memories, that is, those events we need to target within a CBT treatment (e.g. Ehlers and Clark, 2000, Foa et al., 2007). Further, studying symptoms once they are already established tells us little about the neural processes involved in intrusive memory *formation* (aetiology).

### The trauma film paradigm: an experimental psychopathology approach

Electronic media offers a way to use neuroimaging to investigate the brain responses to experimental analogue trauma exposure and intrusive memory formation. Recent work has examined the effects of electronic media, for example television news film footage, on the development of PTSD symptoms. Individuals exposed for prolonged hours to media footage of terrorist attacks have been shown to present higher scores on stress and trauma related symptom scales both a month after the attack (Holman, Garfin, & Silver, 2014) and 2–3 years after the attack (Silver et al., 2013). Additionally, the DSM 5 (American Psychiatric Association, 2013) now includes exposure to trauma through electronic media in the definition of a traumatic event, with the caveat that the exposure is work related. Together, this suggests that traumatic events transmitted through electronic media footage have the potential to induce PTSD-like symptomatology.

The trauma film paradigm is widely used as an experimental analogue of psychological trauma (see Holmes and Bourne, 2008, Lazarus, 1964) and involves healthy participants viewing traumatic footage in line with DSM 5 criteria for a traumatic event (e.g. real life footage depicting actual or threatened death and serious injury; American Psychiatric Association, 2013). The paradigm has been most commonly used in behavioural experiments. Examples include the investigation of cognitive tasks to reduce intrusive memory frequency (e.g. Tetris; Holmes, James, Coode-Bate, & Deeprose, 2009) or vulnerability factors for intrusive memory development (Laposa and Alden, 2008, Wessel et al., 2008).

Recently, we conducted the first study, to our knowledge, to combine the trauma film paradigm with functional Magnetic Resonance Imaging (fMRI) (Bourne et al., 2013; *n* = 22). This provided a prospective measure of the brain activation at the moment of viewing a film scene that would later return as an intrusive memory during the following week. We then replicated this experiment, finding a near identical pattern of results (Clark et al., submitted for publication; *n* = 35). The importance of such replication studies has been particularly noted recently within the field of fMRI (e.g. Carp, 2013, Fletcher and Grafton, 2013).

**Fig. 1:**
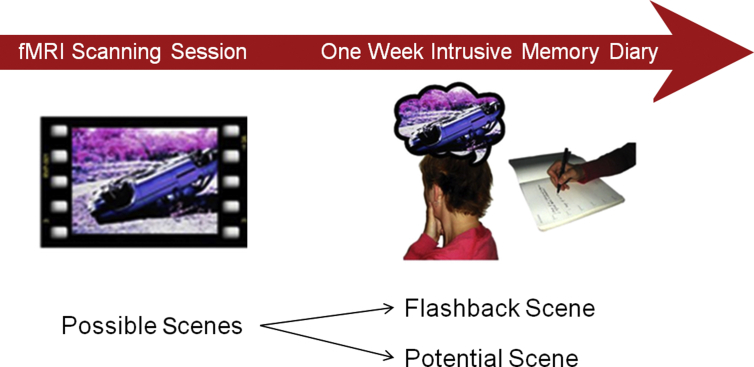
Procedure diagram. Participants viewed traumatic footage while undergoing fMRI. Specific scenes in the film were determined to be ‘Possible’ scenes (scenes that had previously caused intrusive memories in other studies). As intrusive memories are idiosyncratic, Possible scenes became either ‘Flashback’ scenes or ‘Potential’ scenes for each individual. Scene type was determined for each participant retrospectively from the 1 week intrusive memory diaries.

In these studies, unlike most fMRI designs, we could not specify our neuroimaging ‘events’ of interest in advance (i.e. the specific time within stimuli presentation when brain activation is selected to be compared to the rest of stimuli presentation). This is due to intrusive memories being highly idiosyncratic; thus we did not know which scenes in the film would return involuntarily for each individual (just as after a real trauma we do not know which moments will be the hotspots and intrude). The film was created to include 20 scenes that had previously been found to induce intrusive memories. Participants recorded their intrusive memories (defined as mental images of the film content that involuntarily come to mind) for one week in daily life using a pen-and-paper diary. From written descriptions in the intrusive memory diary, intrusions were matched to specific scenes within the film (e.g. the car rolling over the hedge hitting the boy playing football in his garden). Film scenes were then classified on an individual participant basis as either ‘Flashback scenes’ – emotional scenes that returned as an intrusive memory for that individual, or ‘Potential scenes’ – emotional scenes that did not return as an intrusive memory for that individual, but did in other participants (see Fig. 1). On average, 3 of the possible 20 scenes became intrusive memories for each participant; a similar frequency to the number of different events experienced as intrusions after real life trauma (Grey and Holmes, 2008, Holmes et al., 2005).

Using a standard statistical mass univariate regression analysis approach (i.e. the analysis currently most used for fMRI data) we found that Flashback scenes, in comparison to Potential scenes, were characterised by widespread increases in brain activity including the anterior cingulate cortex, thalamus, putamen, insula, amygdala, ventral occipital cortex, left inferior frontal gyrus and bilateral middle temporal gyrus. In brief, brain regions that have previously been associated with emotional processing, visual/mental imagery and memory (see Bourne et al., 2013 for discussion). These results provided, to our knowledge, the first evidence of a ‘neural signature’ at the time of intrusive memory formation.

### Predicting from fMRI; multivariate pattern analysis (MVPA) and machine learning

However, traditional univariate fMRI analysis only highlights an association of peri-traumatic brain responses with later intrusive memories *across a group* of individuals (see for details Jezzard et al., 2001, Smith et al., 2004). Additionally, traditional fMRI analysis relies on the self-report diary to identify the scene type. It would be useful to know the extent to which brain responses during exposure to analogue trauma can actually *predict* a specific moment of the traumatic footage that would later become an intrusive memory, for example, to inform preventative interventions against intrusive memory formation.

Machine learning and multivariate pattern analysis (MVPA) are neuroimaging analysis techniques that can be used to measure prediction accuracy. MVPA makes use of multivariate, spatially extensive patterns of activation across the brain. The patterns of activation across these larger regions can be “learned” through approaches from the field of machine learning. Supervised machine learning techniques optimise input “features” to best separate or describe the two labelled classes of data (i.e. Flashback scene or Potential scene). These “features” are simply summary measures of some aspects of the data. It is through these optimisation steps that machine learning approaches “learn” the patterns that best describe each class of data. Once the patterns have been identified, they can be used to predict the behaviour of new, previously unseen participants. Such approaches can provide greater discriminative ability than spatially localised mass-univariate regression analyses (see for further details, Haxby, 2012, Haynes and Rees, 2006, McIntosh and Mišić, 2013, Mur et al., 2009, Norman et al., 2006). Machine learning can then be used to learn these patterns of activity to accurately predict the occurrence of a new, unseen example of the same event (Lemm et al., 2011, Pereira et al., 2009).

To highlight just a few examples of MVPA techniques applied to fMRI, neural patterns identified by MVPA while participants were exposed to a shock during the presentation of picture stimuli have predicted the later behavioural expression of fear memory (pupil dilation response) between 2 and 6 weeks after encoding (Visser, Scholte, Beemsterboer, & Kindt, 2013). Additionally, MVPA techniques have identified patterns of activation at encoding that can predict later deliberate memory recall (see Rissman & Wagner, 2012).

We hypothesised that machine learning may be able to predict an intrusive memory from just the peri-traumatic brain activation. We aimed first, to investigate whether specific scenes in the film could be identified as later becoming intrusive memories solely from brain activation at the time of viewing traumatic footage by applying machine learning with MVPA. Second, we explore which brain networks are key in MVPA-based prediction of intrusive memory formation, and when the activation of these brain networks in relation to the timing of the intrusive memory scene is important.

## Methods

### Overview

To investigate whether differences in brain activation during the encoding of the trauma film stimuli could predict later intrusive memories of the film, we first trained a machine learning classifier (a support vector machine, SVM) to identify the specific brain activation pattern associated with viewing a film scene that was later involuntarily recalled as an intrusive memory. To do this, the classifier was provided with the timings of the intrusions (from scenes within the original film footage) from the diary data (i.e. from the intrusion content description once we knew which section(s) of the film became an intrusive memory for a given participant). We used a SVM since this approach has been shown to be reliable across multiple studies (see Mourão-Miranda et al., 2005, Pereira et al., 2009). We tested a number of pre-processing and feature generation methods, as these choices have been shown to impact prediction accuracy more consistently than the choice of classifier (Duff et al., 2012, Ku et al., 2008).

Following training, the SVM was used to examine novel data – i.e. brain activation data from a new participant viewing traumatic footage – to pick out the scenes(s) in the footage which would be later experienced as an intrusive memory. Accuracy of prediction was evaluated by the classifiers predictions to those events reported in the participant's diary. Analysis was performed on our previously collected fMRI data (Bourne et al., 2013, Clark et al., submitted for publication).

What is key here is the prediction of which scenes in the film will later return as an intrusive memory in a *new* participant (something even the participant themselves cannot know at this point in time since they have not yet lived the week in which they will experience an intrusive memory). For details of the engineering aspects of the machine learning classifier development we refer the reader to Niehaus et al. (2014).

### Participants

Participants were recruited from the local community separately for the two studies. Twenty-two participants took part in Bourne et al. (2013; mean age = 22 years, SD = 3.08; 17 female), and 35 in Clark et al. (submitted for publication; mean age = 22.43 years, SD = 7.58; 29 female). Inclusion criteria were: participants were aged over 18, had no metal implants, had not taken part in a similar study involving viewing traumatic footage and declared no previous or current psychiatric illness. In the Clark et al. (submitted for publication) study, data could not be analysed for additionally recruited participants where 0 intrusive memories were reported in the diary (*n* = 2) or insufficient performance on a visual recognition memory test (*n* = 1). For 3 further participants the full data was not acquired due to one participant stopping the scan during film viewing, one failing to return to follow up and for one technical issues stopped the scan before film completion. Recruitment material contained information about the potentially distressing content of the film material. Ethical approval was received from NHS Oxfordshire Research Ethics Committee ‘B’ (Bourne et al., 2013) and the University of Oxford Central University Research Ethics Committee (Clark et al., submitted for publication). All participants provided written informed consent and were reimbursed £25 (US $40).

### Data acquisition

fMRI imaging data were acquired on a 3-T Siemens TIM Trio System with a 12-channel head coil [voxel resolution = 3 × 3 × 3 mm^3^; repetition time (TR) = 3 s, echo time (TE) = 30 ms]. T1-weighted structural images were acquired for subject registration using a magnetisation prepared rapid gradient echo (MPRAGE) sequence (voxel resolution = 1 × 1 × 1 mm^3^; TR = 2040 ms; TE = 4.7 ms]. Field maps were obtained for Clark et al. (submitted for publication) with .49 ms echo spacing and 22 ms TE.

### Pre-processing

Data was pre-processed using FEAT (part of FSL – FMRIB's Software Library) version 6.0 (www.fmrib.ox.ac.uk/fsl). Brain extraction was performed using BET (Smith, 2002). High pass filtering was applied with a 100-s cut-off and spatial smoothing with a 5 mm full width half maximum Gaussian kernel. Motion correction was applied with MCFLIRT (Jenkinson, Bannister, Brady, & Smith, 2002). Field map based unwarping was applied to the data from Clark et al. (submitted for publication). Independent Component Analysis (ICA) was performed on all data using MELODIC. Components likely due to noise were removed by the FSL tool FIX. Images were registered to Montreal Neurological Institute (MNI) standard space.

### The machine learning classifier

#### Classifier input features

The raw data from an fMRI study consists of activation levels for each voxel in the brain at every time-point during the study (here, images were captured every 3 s). In order to examine patterns across wider spatial regions, a group level Independent Component Analysis (ICA) was conducted. ICA is a statistical technique that separates the brain signals into independent spatial maps, clustering areas characterised by concurrent activation. This produces independent networks of brain regions that may be activated differentially during different tasks. The group ICA performed here is different to the ICA MELODIC analysis conducted during pre-processing as it identifies regions of concurrent activity across all participants rather than for individual participants (Beckmann & Smith, 2004). Following ICA decomposition, the spatial independent components (ICs) were projected back onto each participant to obtain participant-specific activation levels throughout the spatial region of each IC. The number of ICs was varied to determine the optimal number for predicting flashbacks (detailed in Niehaus et al., 2014). These steps produced a set of activation time-courses for each IC for each participant.

In order to further summarise this data across time, the average level of activation was calculated for three different time periods for each scene type (i.e., for all Flashback and all Potential scenes): the first 6 s of each scene, the remaining duration of the scene, and the 12 s following the conclusion of the scene. In other words, this produced a set of (number of ICs)*(3) values, for each participant, which were used as input features into the machine learning classifiers.

#### Classifier optimisation

**Fig. 2:**
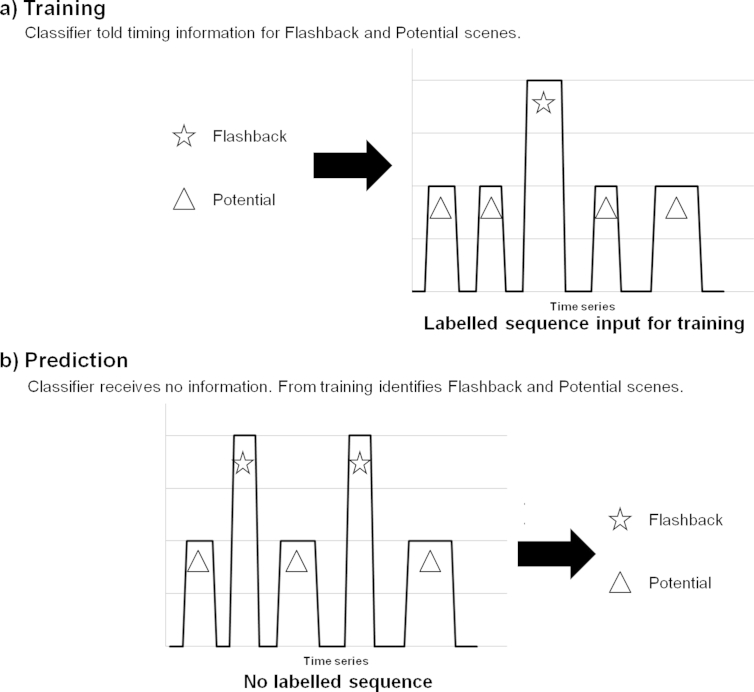
Illustration of the prediction aspect of the machine learning analysis. a. Shows the training element of the machine learning approach. The classifier was provided with information concerning the timing of the Flashback scenes (emotional scenes that returned as a intrusive memory for that individual) and Potential scenes (emotional scenes that did not return as a intrusive memory for that individual, but did in other participants) from which to learn the patterns of brain activation for each scene type. Training was performed on all but 1 participant. b. Shows the predictive element of the machine learning approach. For the 1 participant not included in training the machine learning classifier goes through the brain activation data and attempts to identify the Flashback and Potential scenes.

The support vector machine (SVM) classifier was first optimised on the larger of the 2 data sets (Clark et al., submitted for publication; 35 participants). A labelled sequence of Flashback and Potential scene time points in the film was created from the diaries for each individual participant (as each person may have different intrusions). The input features detailed above, reflecting activation across the brain, were extracted from the fMRI data during these Flashback and Potential time points (see Niehaus et al., 2014 for details). The SVM was then trained on this data to learn the patterns for both scene types, using a leave-one-out methodology to provide a test case: for 1 participant brain activation was not included in the training. Based upon the learned patterns of activity from all other participants, the classifier then attempted to identify the film scenes that later induced intrusive memories for the left-out participant. Identification based on brain activation patterns was the checked against the participant's diary entries (see Fig. 2). This leave-one-out ‘cross-validation loop’ was conducted 35 times, each one with a different participant left out of the training set. Results were averaged over the performance of the SVM on the left-out participant.

Various parameters were examined in order to optimise the predictive ability of the classifier. We compared both linear discriminant analysis and support vector machines as classifiers. Other supervised learning classifiers, such as random forests, could also have been employed, but here we limited our focus for this initial study. Due to the large number of Potential scenes in comparison to the number of Flashback scenes (approximately 5:1), we also compared various balancing techniques. Discussion of classifier optimisation is detailed in Niehaus et al. (2014).

As accuracy alone is not a good indicator of performance within imbalanced data sets (the classifier could achieve high accuracy by always classifying scenes as Potentials) we also assessed sensitivity. We define sensitivity here as the number of true Flashback scenes identified by the classifier out of the total number of Flashback scenes for that participant.

We then tested our ability to predict intrusive memories on our other data set (Bourne et al., 2013; 22 participants). Given our small number of participants, this step was important to test whether prediction performance would generalise to a separate data set.

Finally, we investigated the ability of machine learning to predict intrusive memory formation within a single participant. This within-participant analysis used only those participants within Clark et al. (submitted for publication) that experienced 4 or more different intrusive memories (*n* = 16; mean age 23 years, SD = 7.16; 13 female) leaving one Flashback scene and one Potential scene out for each participant. For within participant analysis, activation levels within individual voxels were used as input features. Voxels were selected with a *t*-test, and brain activity levels were averaged across the entire duration of each scene.

### Identification of brain network functions

Possible functions of the networks identified in the input features (i.e. the ICA components at specific time points), and the names used to describe the cognitive functions of these networks were identified from Smith et al. (2009). Smith et al. (2009) utilised an online repository of published neuroimaging results containing around 30,000 participants from over 1600 published articles (the BrainMap database; Fox and Lancaster, 2002, Laird et al., 2005) to map behavioural tasks (and their proposed corresponding cognitive functions) onto brain regions and networks.

## Results

### Prediction accuracy

In the original training data set the average accuracy of classification within each left-out participant (averaged across the training loops) was 70.1% (SE = 1.8%) with a sensitivity of 60.0% (SE = 5.9%). During replication in the second data set (Bourne et al., 2013); the classifier had a leave-one-out average performance accuracy of 68.0% (SE = 2.4%) and sensitivity of 58.7% (SE = 7.0%). Within a given participant the average accuracy was 97.3% (SE = .93%) and sensitivity of 90.3% (SE = 3.07%).

The best performance for predicting the scenes that would later become intrusive memories was found by using a linear discriminate analysis classifier with 39 independent components. It was found that predictive accuracy significantly decreased when the number of ICs was reduced to below 10 or increased to greater than 70. The best approach for managing the unbalanced class sizes was to apply an increased cost weighting for misclassifying Flashback scenes.

The best performance for predicting which scenes would become intrusive memories within participants was with a support vector machine classifier using 1000 voxels as input features.

### Network identification

**Fig. 3:**
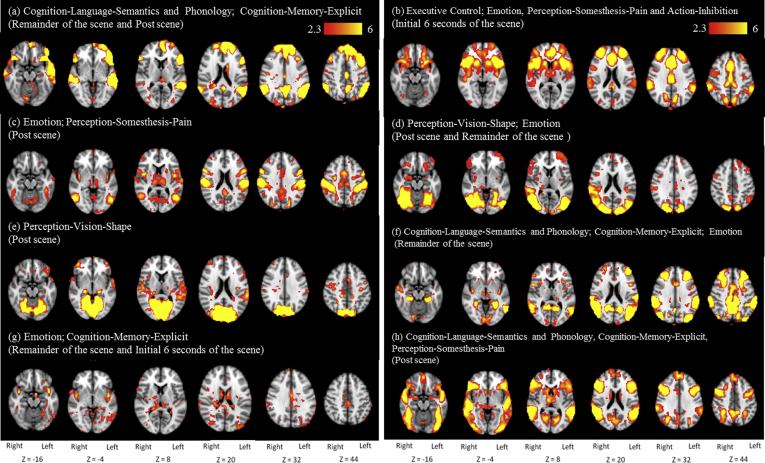
The top weighted input features compromising 8 ICA components (a–h) and their corresponding time points (in brackets) involved in the prediction of a Flashback scene at the time of viewing traumatic footage. The ICA components are presented in the weighted order of the features used in the classifier. Features could be involved at 1 or all of 3 time points; i) the initial 6 s of the Flashback scene, ii) the remainder of the Flashback scene or iii) the 12 s post Flashback scene. Proposed functions of networks within the feature are included to provide a guide to their potential role in intrusive memory formation with names taken from Smith et al. (2009). 6 images are taken for each ICA component and are shown in the axial plane with their corresponding *z* coordinate. The underlying image is the Montreal Neurological Institute (MNI) 152 template, *z*-statistic images are thresholded at *z* > 2.3. *z*-Statistic range is represented by the change in colour.

A total of 117 input features (i.e. averaged activation across the 39 ICA brain networks during the 3 defined time points of the scenes; the initial 6 s of the scene, the remaining duration of the scene after the initial 6 s, and the 12 s post scene) contributed to intrusive memory scene prediction. Below we describe the top weighted input features of the classifier for predicting Flashback versus Potential events (i.e. the features contributing most strongly towards prediction in terms of their weighting within the classifier). We also note their possible cognitive function. While these networks are those top weighted by the classifier, this is not a statistical measure and can only provide a guide towards their predictive contribution. There are 2 components of each feature; the location in the brain (i.e. the ICA component) and the timing of activation. The top weighted input features comprise 8 ICA components, 3 of which were important for intrusive memory prediction at 2 time points (see Fig. 3; ICA components (a–h) are displayed according to their weighting, activation time points are displayed in brackets).

The number of ICA brain networks included in the classifier was restricted so that maximum predictive ability was obtained (increasing from 39 to 70 independent components decreased sensitivity to 47.3%, SE = 7.27). This resulted in relatively widespread brain networks rather than specific brain areas, for which it is harder to attribute a specific function.

The highest weighted input feature (Fig. 3(a)), included the lingual gyrus, left hippocampus, middle temporal cortex, inferior frontal gyrus, supramarginal gyrus, left thalamus, precuneus, middle frontal cortex, left superior frontal cortex and posterior cingulate cortex. Networks within this feature (identified using Smith et al., 2009) have been previously associated with Cognition–Language–Semantics, Cognition–Language–Phonology and Cognition–Memory–Explicit. Activation of this input feature was important for prediction during the remaining duration of the scene (after the initial 6 s) and the 12 s post scene.

The next weighted feature (Fig. 3(b)) included the frontal orbital cortex, insula, frontal, central and parietal operculum, putamen, inferior frontal gyrus, anterior cingulate cortex, thalamus, supramarginal gyrus, middle frontal cortex, pre central cortex and the lateral occipital cortex. Networks within the feature have been associated with a number of functions termed ‘Executive Control’ in addition to Emotion, Perception–Somesthesis–Pain and Action–Inhibition (Fig. 3(b)). Activation of the feature was important for prediction during the initial 6 s of the scene.

The third weighted feature (Fig. 3(c)), involved the thalamus, insula, central and parietal operculum, putamen, inferior frontal gyrus and the anterior and posterior cingulate cortex. Networks in these areas have been associated with Emotion and Perception–Somesthesis–Pain. The feature was predictive in the 12 s post scene.

The fourth weighted feature (Fig. 3(d)) involved the lateral occipital cortex, occipital fusiform, amygdala, right putamen, right inferior frontal gyrus, right insula, right thalamus and occipital pole. Networks in the feature have been associated with Perception–Vision–Shape and Emotion. Activation levels were important for prediction during the remaining duration of the scene (after the initial 6 s) and the 12 s post scene.

The fifth feature (Fig. 3(e)) predominantly involved occipital fusiform gyrus, temporal occipital fusiform gyrus, lateral occipital cortex, occipital pole and intracalcarine cortex. This network has been associated with Perception–Vision–Shape. Activation of the feature was important for prediction in the 12 s post scene.

The sixth weighted feature (Fig. 3(f)) involved a wide range of regions including the parahippocampal gyrus, middle temporal cortex, right hippocampus, insula, thalamus, lingual gyrus, occipital pole, putamen, precuneus, frontal operculum, middle frontal cortex, left inferior frontal gyrus, angular gyrus, lateral occipital cortex, supramarginal gyrus and the anterior and posterior cingulate. Networks involved have been associated with Cognition–Language–Semantics, Cognition–Language–Phonology, Cognition–Memory–Explicit, Emotion, and the Default Mode Network. Activation of the feature was important for prediction during the remaining duration of the scene (after the initial 6 s).

The seventh weighted feature (Fig. 3(g)) involved the insula, left parahippocampus, left hippocampus left middle temporal cortex, planum polare (part of Wernicke's area), thalamus, posterior cingulate cortex and lateral occipital cortex. Networks have been associated with Emotion and Cognition–Memory–Explicit. Activation was important during the initial 6 s of the scene and the remaining duration of the scene (after the initial 6 s).

The final feature shown here (Fig. 3(h)) involved the lateral occipital cortex, amygdala, thalamus, accumbens, putamen, frontal operculum, inferior frontal gyrus, supramarginal gyrus, superior and middle frontal cortices, and the precuneus. Networks have been associated with Cognition–Language–Semantics, Cognition–Language–Phonology, Cognition–Memory–Explicit and Perception–Somesthesis–Pain. Activation of the feature was important for prediction in the 12 s post scene.

## Discussion

Intrusive memories are a target in CBT for Post-Traumatic Stress Disorder. This paper has presented an experimental psychopathology approach to understanding the underlying neural mechanisms of intrusive memories using recent advances in brain imaging analysis techniques. Here we show that intrusive memory formation (that is, which moments within an analogue trauma will be spontaneously recalled in the week after viewing the trauma) can be *predicted* solely from brain activation at the time of viewing the traumatic film footage. Intrusive memories are highly idiosyncratic; on average 3 of a possible 20 scenes within the trauma film returned as an intrusive memory, but which 3 varied according to each individual. The machine learning (Support Vector Machine) classifier, using MVPA for the input variables, was able to predict the later occurrence of a specific intrusive memory in an unseen participant from the data set with 70.1% accuracy and 60% sensitivity. This generalised to a novel data set of new participants with 68% accuracy and 58.7% sensitivity showing good replication. Further, we could predict intrusive memory development within a given participant with 97% accuracy and 90.3% sensitivity (i.e. if we know someone's brain reaction associated with intrusive memory development within the trauma trained on all their intrusions except one, we could then accurately predict a new example of intrusive memory formation – the missing intrusion).

These results provide support for a hypothesised ‘intrusive memory signature’ within brain activation at the time of the original analogue trauma encoding (Bourne et al., 2013, Clark et al., submitted for publication. Our results suggest that not only is brain activation at encoding *associated* with the occurrence of intrusive memories, but we can measure the accuracy with which brain activity can be used to *predict* specific scenes that will become intrusive memories. That is, a specific pattern of brain response during trauma exposure contributes to determining if a certain moment during the trauma will be later re-experienced as an intrusive memory or not. A related effect has previously been noted in the non-clinical memory literature, called the subsequent memory effect (Dobbins and Wagner, 2005, Paller and Wagner, 2002, Rissman and Wagner, 2012) albeit for non-intrusive types of memory.

Our data indicate a number of brain networks where analogue peri-traumatic activation appears crucial for intrusive memory prediction. The networks used by the machine learning classifier for intrusive memory prediction are in line with neurocircuitry models of PTSD patients (Admon et al., 2013, Rauch et al., 2006): hyper-responsivity in the amygdala and associated limbic regions involved in emotional processing and the dorsal anterior cingulate cortex have been found in PTSD samples. These regions are also active in the networks implicated in the current machine learning analysis. In particular, increased activation in emotional processing regions was involved in 5 of our 8 top weighted networks used to predict intrusive memory formation after analogue trauma. Findings are in line with fMRI results for pre-disposing factors for later clinical PTSD symptom development (see Admon et al., 2013).

Interestingly, both our univariate and multivariate analyses highlight the involvement of possible language related networks in intrusive memory formation. This is interesting clinically since early Positron Emission Tomography (PET) studies on Vietnam veterans revealed decreased activation in Broca's area (Shin et al., 1997, Shin et al., 1999). As cognitive behavioural therapies are language based, further understanding of the involvement of language in intrusive sensory memory development may be relevant to optimising therapeutic interventions. Additionally, it may help us to experimentally explore why some early aftermath counselling interventions, such as critical incident stress debriefing, have been found to be harmful (Roberts et al., 2009, Rose et al., 2002).

Overall, our results suggest that we were able to so-called ‘mind read’ (Norman et al., 2006), or in more literal terms decode the brain activity during film viewing to identify which scenes of the film would later intrude. This new approach of using machine learning and MVPA strengthens our understanding of neural mechanisms underpinning intrusive memory formation with clinical relevance. At a general process level we can derive information from the specific brain networks predictive of intrusive memories, suggesting which cognitive functions may be most relevant for intrusive memory formation, and present possible mechanistic targets for preventative interventions. Additionally, differences at an individual level may open future possibilities of early screening for risk of PTSD development in the immediate aftermath of trauma for targeted early intervention. A trauma film paradigm with fMRI might even be developed for use prior to real trauma exposure for identifying those who may be more vulnerable to trauma generally (e.g. within army recruits or emergency personnel).

### Future work applying machine learning and fMRI to clinical psychology more broadly

How else may we be able to use advanced neuroimaging techniques within clinical psychology? MVPA predictive techniques may be able to use neuroimaging data to predict (among others) likelihood of illness occurrence in at-risk groups. For example, in depression, meta-analysis of fMRI studies indicates abnormal activity across various brain regions (e.g. amygdala, dorsal anterior cingulate cortex, insula) in depressed patients compared to healthy controls in response to negative stimuli (Hamilton et al., 2012). Machine learning classifiers have been able to utilise these differences to predict whether participants are grouped as patients or healthy controls solely from differences in brain activity at the time of viewing sad faces (Fu et al., 2008). Extending this to at-risk groups may help target resources and treatments, and possibly in the future could even aid diagnosis. Above, for example, we have suggested how our line of enquiry could be developed to aid identification those of at risk for PTSD, e.g. in emergency personnel.

Cognitive Bias Modification (CBM) is a procedure which aims to retune dysfunctional attentional and emotional biases (e.g. Browning et al., 2010, Mathews and MacLeod, 2005, Niles et al., 2013, Waters et al., 2013). However, we lack objective methods to test whether an individual has altered their cognitive bias. If machine learning were able to classify cognitive biases it may be possible for the therapist to objectively observe whether a patient is able to modulate and reduce a cognitive bias by observing alterations in the underpinning brain response. Future studies could readily apply work to this area given the ease of studying cognitive bias modification during fMRI (Browning, Holmes, Murphy, Goodwin, & Harmer, 2010).

Further work using MVPA and machine learning may be able to identify brain activity at an individual participant level. Understanding the presentation of symptoms at an individual level may help assess the effects of a treatment for that patient by performing neuroimaging before and after treatments (e.g. exposure based therapy; Foa et al., 2007). MVPA techniques could compare brain response to trauma related stimuli, hypothesising that successful treatment would be signalled by a change in brain activation patterns compared to pre-treatment in those specific networks that were predictive of intrusive memory formation (e.g. as in Kriegeskorte, 2009, Kriegeskorte, 2011). This may also be applicable to fear extinction and return of fear; while initial fear extinction is relatively easy to induce, ensuring that the extinction remains permanent is more difficult (Vervliet, Craske, & Hermans, 2013). MVPA utilising the brain activations involved in extinction (e.g. recruitment of the ventromedial prefrontal cortex and hippocampus; Milad et al., 2007) may be able to suggest whether a fear memory has undergone permanent extinction.

Advanced neuroimaging techniques may provide an avenue to overcome the occasional limitations of subjective reports of symptomatology, such as in patients who are mute, or difficulties that some patients have with verbally describing their precise symptoms. For example, work outside of clinical psychology has demonstrated the potential of MVPA to identify a specific image seen by a participant undergoing fMRI (Kay, Naselaris, Prenger, & Gallant, 2008). After examining the brain activity associated with viewing neutral images (picture stills), of which the content was known to the computer model, the model was able to pick out, from a large set of new picture stimuli, which specific image was seen by the participant. More recently, this technique was extended to film stimuli, following the same procedure but using dynamic neutral movies (Nishimoto et al., 2011). Further, by comparing brain activity identified to specific visual content and the brain activity during sleep, it has been possible to describe the content of a participant's dream (Horikawa, Tamaki, Miyawaki, & Kamitani, 2013).

### Methodological and conceptual limitations

Viewing traumatic film footage is not the same as experiencing an actual trauma and findings need to be extended to clinical samples. However, we note that intrusive memories experienced after traumatic events and intrusive memories in everyday life (such as those in our experimental procedure) can be considered on a continuum (Kvavilashvili, 2014). Additionally, PTSD symptoms have been reported following exposure to traumatic media footage (Holman et al., 2014, Silver et al., 2013). Changes to DSM 5 (American Psychiatric Association, 2013) now include exposure to traumatic content via electronic media (e.g. films) as sufficient for a diagnosis when the exposure is work related which suggests, at least at times, film footage can create real PTSD symptoms.

Additionally, we note that our study has other limitations. The number of participants was limited, reducing the extent it was possible to test different machine learning strategies. The unusual scarce-event study design meant that it was nevertheless crucial to test and optimise pre-processing and feature generation approaches on the first study participants, and then test the optimised approach on the independent sample.

Links between brain activations and cognitive function have been made here with what is termed in the fMRI literature as ‘reverse inference’, i.e. a brain region is identified as being predictive of a later event (e.g. an intrusive memory); in other studies that region was active when participants were performing a task engaging a particular cognitive process; it is therefore likely that this cognitive process is involved in intrusive memory formation (see Poldrack, 2006, Poldrack, 2011). However, the problem arises as to the specificity of the identified region in that specific cognitive process – it is unlikely that a brain region has just one cognitive function. On the other hand, we note that the predictive capabilities of the machine learning are not in question, only potential interpretations of the brain regions involved.

Finally, the DSM-5 distinguishes between intrusive memories and dissociative ‘flashbacks’. Dissociation has been studied previously in behavioural experiments using the trauma film paradigm (e.g. Hagenaars and Krans, 2010, Hagenaars et al., 2008, Holmes et al., 2004). However, no measure of dissociation was taken in the current study and thus we could not examine any possible effects of dissociation to the current work. A continuum has been proposed ranging from involuntary autobiographical memories in everyday life to recurrent intrusive memories and in the most extreme (and rarest) form dissociative flashbacks (Kvavilashvili, 2014). Investigating dissociation in combination with fMRI is therefore an important step for future work (e.g. Daniels et al., 2012).

## Conclusions

Using machine learning and MVPA on fMRI data of trauma film encoding, we have demonstrated that peri-traumatic brain activation is able to *predict* moments that would later return as an intrusive memory with 68% accuracy across participants and within a given participant with 97% accuracy. Here, we make an attempt to import ideas from basic neuroscience to contribute to an area of mental health – intrusive trauma memories. We suggest certain advance neuroimaging techniques may even be developed for use in studying relatively infrequently occurring and idiosyncratic events in mental health symptomatology (such as intrusive memories) and be used to predict individual's future symptom response.
